# COVID-19 vaccination status, side effects, and perceptions among breast cancer survivors: a cross-sectional study in China

**DOI:** 10.3389/fpubh.2023.1119163

**Published:** 2023-04-17

**Authors:** Yali Xu, Linrong Li, Xiaomeng Li, Haolong Li, Yu Song, Yongmei Liu, Chang Chen, Haoting Zhan, Zhe Wang, Xinxin Feng, Mohan Liu, Yingjiao Wang, Guanmo Liu, Yang Qu, Yuechong Li, Yongzhe Li, Qiang Sun

**Affiliations:** ^1^Department of Breast Surgery, Peking Union Medical College Hospital, Peking Union Medical College, Chinese Academy of Medical Sciences, Beijing, China; ^2^Department of Clinical Laboratory, State Key Laboratory of Complex Severe and Rare Diseases, Peking Union Medical College Hospital, Chinese Academy of Medical Science and Peking Union Medical College, Beijing, China; ^3^Department of Medical Research Center, Peking Union Medical College Hospital, Chinese Academy of Medical Science and Peking Union Medical College, Beijing, China

**Keywords:** COVID-19 pandemic, vaccine, side effects, breast cancer, SARS-CoV-2

## Abstract

**Introduction:**

Breast cancer is the most prevalent malignancy in patients with coronavirus disease 2019 (COVID-19). However, vaccination data of this population are limited.

**Methods:**

A cross-sectional study of COVID-19 vaccination was conducted in China. Multivariate logistic regression models were used to assess factors associated with COVID-19 vaccination status.

**Results:**

Of 2,904 participants, 50.2% were vaccinated with acceptable side effects. Most of the participants received inactivated virus vaccines. The most common reason for vaccination was “fear of infection” (56.2%) and “workplace/government requirement” (33.1%). While the most common reason for nonvaccination was “worry that vaccines cause breast cancer progression or interfere with treatment” (72.9%) and “have concerns about side effects or safety” (39.6%). Patients who were employed (odds ratio, OR = 1.783, *p* = 0.015), had stage I disease at diagnosis (OR = 2.008, *p* = 0.019), thought vaccines could provide protection (OR = 1.774, *p* = 0.007), thought COVID-19 vaccines were safe, very safe, not safe, and very unsafe (OR = 2.074, *p* < 0.001; OR = 4.251, *p* < 0.001; OR = 2.075, *p* = 0.011; OR = 5.609, *p* = 0.003, respectively) were more likely to receive vaccination. Patients who were 1–3 years, 3–5 years, and more than 5 years after surgery (OR = 0.277, *p* < 0.001; OR = 0.277, *p* < 0.001, OR = 0.282, *p* < 0.001, respectively), had a history of food or drug allergies (OR = 0.579, *p* = 0.001), had recently undergone endocrine therapy (OR = 0.531, *p* < 0.001) were less likely to receive vaccination.

**Conclusion:**

COVID-19 vaccination gap exists in breast cancer survivors, which could be filled by raising awareness and increasing confidence in vaccine safety during cancer treatment, particularly for the unemployed individuals.

## Introduction

Coronavirus disease 2019 (COVID-19) is taking a huge toll on the people and healthcare system of China and the rest of the world (1). As of July 30, 2022, 229,510 confirmed cases and 5,526 deaths were reported in the Chinese mainland (2), and 557,917,904 confirmed cases and 6,358,899 deaths were reported globally (3).

Of specific interest are patients with breast cancer because of high prevalence, high mortality rate, (4–7) and potential immunosenescence to vaccination in this population (8–11). As the most common cancer and the fifth leading cause of cancer mortality worldwide, (12, 13) breast cancer is the most prevalent malignancy in the population diagnosed with COVID-19 (4). During the prevaccination phase from February 27, 2020 to November 30, 2020, the 28-day case fatality rate (CFR_28_) of COVID-19 was 13.9% among patients with breast cancer (14).

Periodic vaccination is expected to be an effective solution. It was reported that vaccinated patients diagnosed with breast cancer achieved an improved CFR_28_ and reduced COVID-19 severity compared with unvaccinated controls (14). The National Comprehensive Cancer Network (NCCN) recommended patients with breast cancer receive COVID-19 vaccination as soon as possible. Patients with breast cancer under active treatment or not were prioritized for a third dose of mRNA vaccines within 1 year of the initial vaccine administration (15). However, safety reports and acceptance of COVID-19 vaccines in patients with breast cancer were limited, resulting in vaccine hesitancy and policy delay.

In this population-based survey study, we investigated the vaccination status, side effects, and perceptions among breast cancer survivors during the COVID-19 pandemic. To our knowledge, this is the largest cross-sectional study on COVID-19 vaccination in the breast cancer population. The findings of this study would help recognize the current COVID-19 vaccination status in the breast cancer population, and provide evidence for customizing strategies to promote vaccination globally.

## Methods

### Study population

Data were collected through a nonprobability online survey between May 22 and July 9, 2022.

We recruited patients who were older than 18 years, pathologically diagnosed with breast cancer, and underwent breast surgery at Peking Union Medical College Hospital (PUMCH), Beijing, China between 2010 and 2022. Participants who did not reside in the Chinese mainland (e.g., Hong Kong Special Administrative Region, Macao Special Administrative Region, and Taiwan Province), and those with documented severe cognitive impairment were excluded. Patients were quota-sampled to match the respective population (Chinese breast cancer population) distributions for age (by both incidence and prevalence) and years after surgery. The survey was conducted using a self-administered questionnaire *via* a web-based investigation platform Wenjuanxing.^1^ Potential participants can fill in the survey after receiving an invitation to participate *via* the telephone or WeChat (a free social communication application with more than 1.2 billion active users in China). The questionnaire consists of 37 questions on sociodemographic characteristics, health and disease status, COVID-19 pandemic, and vaccination (Supplementary file 1). A pilot study had been conducted before the formal initiation of the study. The questionnaire’s content was refined based on feedback from 30 participants, with an average time of 5.8 min taken to complete the questionnaire. The response rate was not available, neither were the characteristics of the nonresponders because of the recruitment methods. Information confidentiality was guaranteed to each participant. Data were accessed and analyzed by members of the research team.

### Variables

The survey assessed numerous sociodemographic variables of the participants, including educational attainment, monthly household income, administrative regions, rurality, work status, and having children under 18 years of age. Furthermore, the questionnaire variables related to health and disease status were assessed, including self-perceived health, recent breast cancer-related treatment, time of surgery, history of food or drug allergies, and history of other vaccine allergies. The questionnaire submission time was automatically recorded by the platform, and the time after surgery was obtained by calculating the period between the questionnaire submission time and the time of surgery. Participants were asked to voluntarily give their identification numbers registered at PUMCH to minimize the time required to complete the questionnaire and improve accuracy. Variables, including age, gender, and time of surgery were attained and validated using the identification number by referring to the hospital information system (HIS) of PUMCH. Additionally, the participants’ clinical stage at diagnosis, histology, histological grade, and molecular subtype were determined by referring to the participants’ pathological reports of surgical specimens from HIS in accordance with the Chinese Society of Clinical Oncology and NCCN guidelines (16, 17). Ki67 values of 20% and more were considered high.

Variables related to the COVID-19 pandemic and vaccination were assessed, including history of COVID-19 infection and vaccination status. Furthermore, participants were asked whether they were worried about COVID-19 infection. They were also asked whether they had a former experience in consulting healthcare workers about COVID-19 vaccination, and, if any, whether the questions were answered. Participants who had not been vaccinated were asked to provide reasons for nonvaccination. Other reasons, apart from the choice options, were allowed. Participants who had been vaccinated were asked about the time, type, and side effects of each dose, as well as the main reason for and the main concern before vaccination. Participants were asked to check their vaccine records before filling in the questionnaire to ensure accuracy of the self-reported information. Additionally, participants were asked whether they believed vaccines could prevent COVID-19 and to what extent did they believe the COVID-19 vaccines are safe. Finally, fully or partially immunized participants were asked whether, if possible, they were willing to receive another dose of COVID-19 vaccine. Participants who answered no were asked to provide reasons.

### Statistical methods

Data cleaning was performed using Microsoft Excel 2016 version 15.27 (Microsoft Corporation, Redmond, WA, USA) and R software version 4.1.1 (R Foundation for Statistical Computing, Vienna, Austria) (18). Descriptive statistics were performed to summarize participants’ characteristics using IBM SPSS Statistics version 26 (IBM Corporation, Armonk, NY, USA) (19). Continuous variables were described using median and interquartile range (IQR) after performing the Shapiro–Wilk test, showing skewness distribution, or using mean and standard deviation given symmetric distribution. Variables were compared among different subgroups using a *t*-test, one-way analysis of variance, or Wilcoxon rank-sum test when appropriate. Categorical variables were reported as percentages, and variables were compared among different subgroups using Pearson’s chi-squared test. Or Fisher’s exact test was performed when one or more of the cell counts in an R × C table was <5.

Univariate and multivariate binary logistic regression analyses were performed to explore potential and independent variables associated with vaccination status using IBM SPSS Statistics. Vaccinated participants (*Y* = 1) were a combination of 1,459 participants, who had been administered with one, two, or three doses of vaccines. While nonvaccinated participants (*Y* = 0) were 1,445 participants. The variables included in logistic regression analyses were chosen based on previous studies and *a priori* discussion by the research team (20–22). For the multivariate logistic regression analyses, forward stepwise (likelihood ratio) selection was used to eliminate variables with a value of *p* ≥ 0.05 to arrive at the final model. The goodness of fit for the multivariable logistic model with procession was tested using the Hosmer–Lemeshow test. Missing indicators were used to represent missing values in the model. The odds ratio (OR), 95% confidence intervals (CI), and value of *p* were calculated. A two-sided value of *p* < 0.05 was considered statistically significant.

## Results

### Participant characteristics

A total of 2,915 participants completed the questionnaire. Among them, six who did not reside in the Chinese mainland were excluded, together with five duplicates. Therefore, the final analysis included 2,904 participants. All participants were female. Some characteristics, such as regional distributions, differed, whereas age distribution was comparable with the Chinese breast cancer population, and years after surgery were balanced (Supplementary file 2).

Participants’ age ranged from 25 to 95 years (median = 51, IQR = 14). More than half of the participants (51.3%) had a bachelor’s degree or higher, 61.2% reported a monthly household income of more than 5,000 yuan *per capita*, 37.2% had children under 18 years of age, 43.3% were employed, and 27.1% had lived with breast cancer for more than 5 years. No participants had metastatic disease at diagnosis, 56.2% had invasive ductal carcinoma, and 54.3% had luminal subtypes. Furthermore, 98.2% thought their health status was general or good, and 76.2% recently underwent breast cancer-related treatments, including 28.4% underwent chemotherapy, radiotherapy, or targeted therapy (Table 1).

**Table 1:** Basic characteristics of breast cancer survivors.

	Total sample (*N* = 2,904) *n* (col%)	Vaccinated participants (*n* = 1,459) *n* (col%)	Not vaccinated participants (*n* = 1,445) *n* (col%)	Value of *p*
**Sociodemographic variables**				
Age in years				
25–39	312 (10.7)	162 (11.1)	150 (10.4)	0.202
40–49	836 (28.8)	439 (30.1)	397 (27.5)	
50–59	900 (31.0)	450 (30.8)	450 (31.1)	
60–69	457 (15.7)	229 (15.7)	228 (15.8)	
70–79	131 (4.5)	57 (3.9)	74 (5.1)	
80+	36 (1.2)	13 (0.9)	23 (1.6)	
Missing^*^	232 (8.0)	109 (7.5)	123 (8.5)	
**Educational attainment**				
Undergraduate	1,162 (40.0)	599 (41.1)	563 (39.0)	0.038
Postgraduate	327 (11.3)	180 (12.3)	147 (10.2)	
High school and below	1,415 (48.7)	680 (46.6)	735 (50.9)	
**Monthly household income *per capita*, yuan**				
2,000–5,000	947 (32.6)	446 (30.6)	501 (34.7)	0.065
<2000	181 (6.2)	88 (6.0)	93 (6.4)	
5,000-10,000	973 (33.5)	497 (34.1)	476 (32.9)	
>10,000	803 (27.7)	428 (29.3)	375 (26.0)	
**Administrative regions**				
East	242 (8.3)	122 (8.4)	120 (8.3)	0.448
North	2,246 (77.3)	1,119 (76.7)	1,127 (78.0)	
Northeast	230 (7.9)	123 (8.4)	107 (7.4)	
Central	77 (2.7)	40 (2.7)	37 (2.6)	
South	25 (0.9)	17 (1.2)	8 (0.6)	
Southwest	21 (0.7)	8 (0.5)	13 (0.9)	
Northwest	63 (2.2)	30 (2.1)	33 (2.3)	
**Living area**				
Urban	2,709 (93.3)	1,360 (93.2)	1,349 (93.4)	0.023
Rural	195 (6.7)	99 (6.8)	96 (6.6)	
Work status				
Unemployed	307 (10.6)	149 (10.2)	158 (10.9)	<0.001
Employed	1,257 (43.3)	707 (48.5)	550 (38.1)	
Retired	1,337 (46.0)	601 (41.2)	736 (50.9)	
Student	3 (0.1)	2 (0.1)	1 (0.1)	
**Have children under age 18**				
No	1824 (62.8)	891 (61.1)	933 (64.6)	0.051
Yes	1,080 (37.2)	568 (38.9)	512 (35.4)	
**Health and disease status**				
**Self-perceived health**				
Bad	53 (1.8)	35 (2.4)	18 (1.2)	0.008
General	702 (24.2)	327 (22.4)	375 (26.0)	
Good	2,149 (74.0)	1,097 (75.2)	1,052 (72.8)	
**Recent breast cancer-related treatment**				
Cytotoxic therapy^**^	826 (28.4)	495 (33.9)	331 (22.9)	<0.001
Endocrine therapy	1,298 (44.7)	541 (37.1)	757 (52.4)	
Traditional Chinese medicine	90 (3.1)	38 (2.6)	52 (3.6)	
No treatment	662 (22.8)	372 (25.5)	290 (20.1)	
Missing^*^	28 (1.0)	13 (0.9)	15 (1.0)	
**Time after surgery**				
<1 year	585 (20.1)	426 (29.2)	159 (11.0)	<0.001
1–3 years	916 (31.5)	379 (26.0)	537 (37.2)	
3–5 years	583 (20.1)	256 (17.5)	327 (22.6)	
> = 5 years	787 (27.1)	375 (25.7)	412 (28.5)	
Missing^*^	33 (1.1)	23 (1.6)	10 (0.7)	
**History of food or drug allergies**		23		
No	2,260 (77.8)	1,214 (83.2)	1,046 (72.4)	<0.001
Yes	644 (22.2)	245 (16.8)	399 (27.6)	
**History of other vaccine allergies**				
No	2,769 (95.4)	1,425 (97.7)	1,344 (93.0)	<0.001
Yes	135 (4.6)	34 (2.3)	101 (7.0)	
**Stage at diagnosis**				
0	165 (5.7)	74 (5.1)	91 (6.3)	<0.001
I	589 (20.3)	426 (29.2)	163 (11.3)	
II	662 (22.8)	317 (21.7)	345 (23.9)	
III	515 (17.7)	224 (15.4)	291 (20.1)	
IV	0	0	0	
Missing^*^	973 (33.5)	418 (28.6)	555 (38.4)	
**Histology**				
Carcinoma *in situ*	246 (8.5)	132 (9.0)	114 (7.9)	0.665
Invasive ductal carcinoma	1,633 (56.2)	830 (56.9)	803 (55.6)	
Invasive lobular carcinoma	74 (2.5)	42 (2.9)	32 (2.2)	
Others	170 (5.9)	88 (6.0)	82 (5.7)	
Missing^*^	781 (26.9)	367 (25.2)	414 (28.7)	
**Histological grade**				
G1	213 (7.3)	103 (7.1)	110 (7.6)	0.648
G2	1,041 (35.8)	535 (36.7)	506 (35.0)	
G3	590 (20.3)	307 (21.0)	283 (19.6)	
Missing^*^	1,060 (36.5)	514 (35.2)	546 (37.8)	
**Molecular subtype**				
Luminal A	491 (16.9)	254 (17.4)	237 (16.4)	0.685
Luminal B	1,085 (37.4)	528 (36.2)	557 (38.5)	
HER2 over-expression subtype	153 (5.3)	77 (5.3)	76 (5.3)	
Basal-like	183 (6.3)	94 (6.4)	89 (6.2)	
Missing^*^	992 (34.2)	506 (34.7)	486 (33.6)	
**Variables related to COVID-19**				
**History of COVID-19 infection**				
No	2,889 (99.5)	1,451 (99.5)	1,438 (99.5)	0.987
Yes, no symptoms	2 (0.1)	1 (0.1)	1 (0.1)	
Yes, mild symptoms	6 (0.2)	3 (0.2)	3 (0.2)	
Yes, severe symptoms	7 (0.2)	4 (0.3)	3 (0.2)	
**Worried about infection**				
No	867 (29.9)	422 (28.9)	445 (30.8)	0.270
Yes	2037 (70.1)	1,037 (71.1)	1,000 (69.2)	
**Have you consulted healthcare workers about COVID-19 vaccines?**				
No	1,123 (38.7)	571 (39.1)	552 (38.2)	0.046
Yes, my questions were answered.	1,263 (43.5)	653 (44.8)	610 (42.2)	
Yes, my questions were not answered.	518 (17.8)	235 (16.1)	283 (19.6)	
**Think vaccines can provide protection**				
No	557 (19.2)	231 (15.8)	326 (22.6)	<0.001
Yes	2,347 (80.8)	1,228 (84.2)	1,119 (77.4)	
**Perceptions on vaccine safety**				
General	1,206 (41.5)	493 (33.8)	713 (49.3)	<0.001
Safe	1,114 (38.4)	605 (41.5)	509 (35.2)	
Very safe	250 (8.6)	168 (11.5)	82 (5.7)	
Not safe	249 (8.6)	127 (8.7)	122 (8.4)	
Very unsafe	85 (2.9)	66 (4.5)	19 (1.3)	

Values in red indicates these are statistically significant.

* Missing values were not included for statistical analysis.

** Chemotherapy/radiotherapy/targeted therapy, with/without endocrine therapy or traditional Chinese medicine.

### COVID-19 vaccination status and underlying reasons

**Figure 1:**
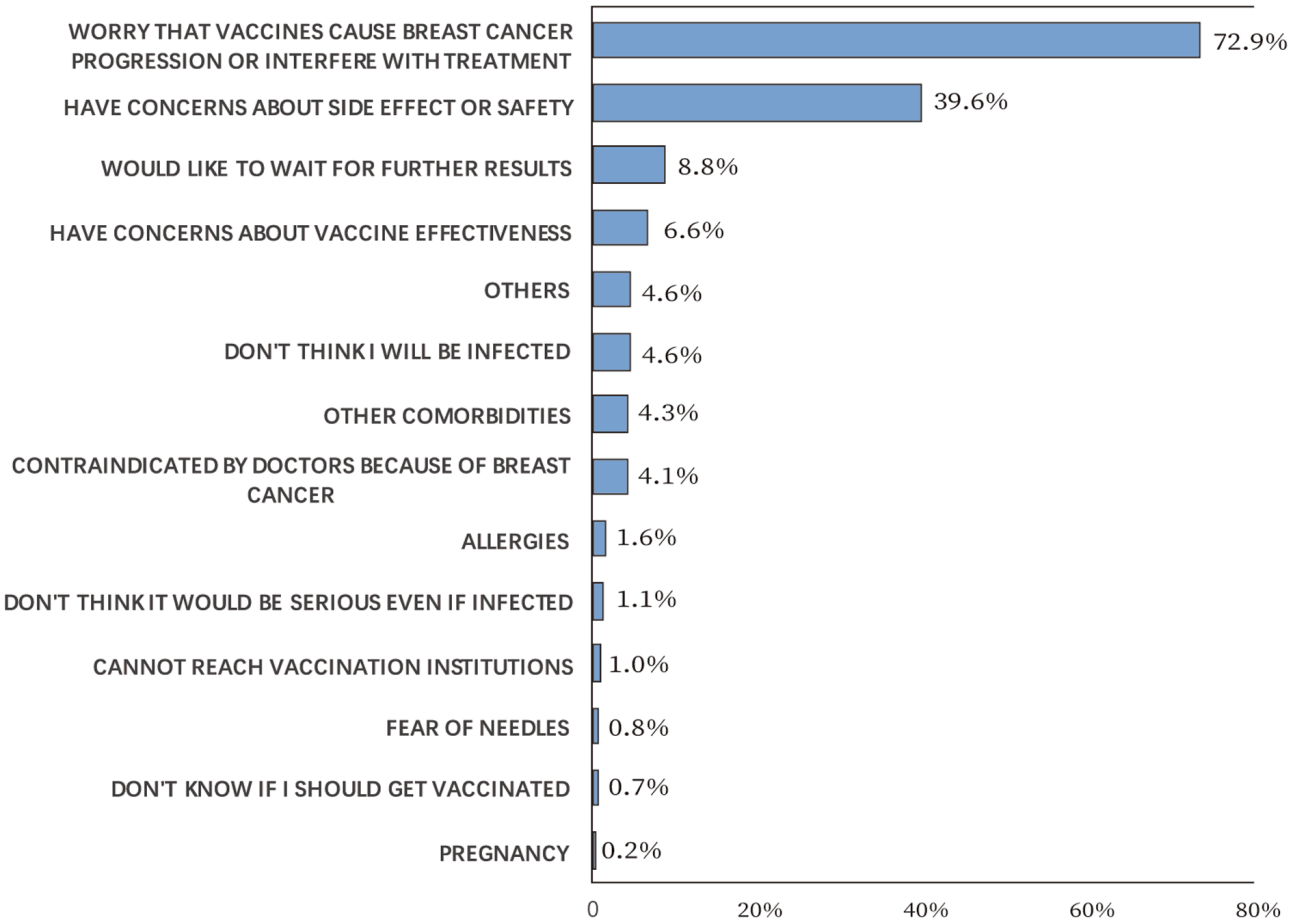
Reasons for non-vaccination.

**Figure 2:**
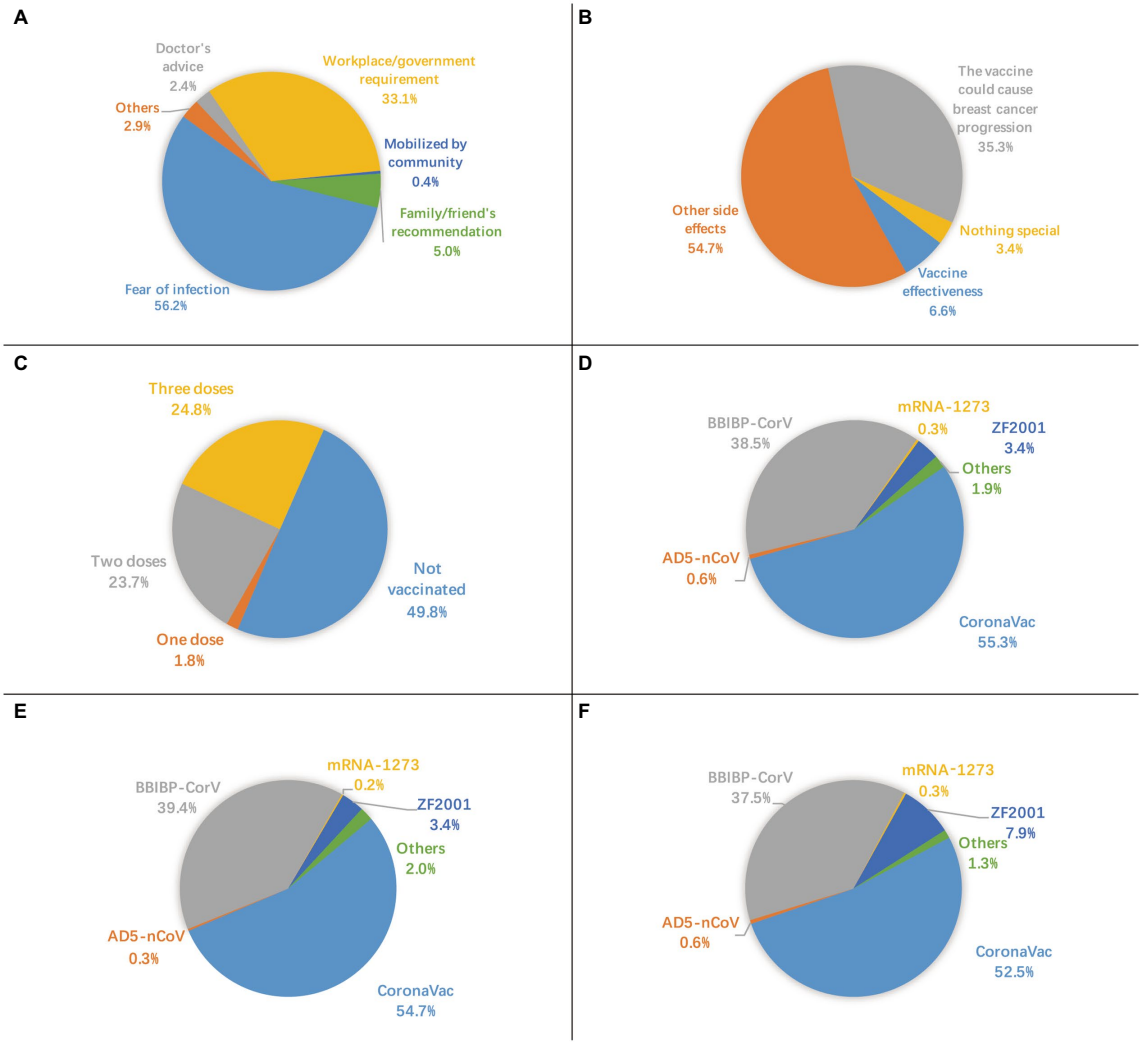
**(A)** main reason for vaccination, **(B)** main concern before vaccination, **C** status of vaccination, **D** type of first vaccine dose, **E** type of second vaccine dose, **F** type of third vaccine dose.

Of the 2,904 survey participants, 99.5% had no history of COVID-19 infection, though 70.1% were worried about infection. A total of 15 participants had a history of COVID-19 infection, of them seven participants had not been vaccinated. The COVID-19 vaccination coverage rate was 50.2%. Reasons for nonvaccination are shown in Figure 1. The most common reason was “worry that vaccines cause breast cancer progression or interfere with treatment,” accounting for 72.9%, followed by “have concerns about side effects or safety,” accounting for 39.6% of nonvaccinated participants. The most common main reason for vaccination was “fear of infection,” accounting for 56.2%, followed by “workplace/government requirement,” accounting for 33.1% of the vaccinated participants (Figure 2A). Furthermore, for vaccinated participants, “the vaccine could cause breast cancer progression” represented the second leading main concern before vaccination (35.3%), following “other side effects” (54.7%; Figure 2B).

In total, 1.8% (52/2,904) of the participants received one dose, 23.7% (687/2,904) received two doses, and 24.8% (720/2,904) received three doses (Figure 2C). Inactivated virus vaccines, including BBIBP-CorV (Sinopharm’s Beijing Institute of Biological Products), CoronaVac (Sinovac Biotech), KCONVAC (Shenzhen Kangtai Biological Products), and WIBP-CorV (Sinopharm’s Wuhan Institute of Biological Products), were used in 94.8, 95.3, and 90.4% of the first, second, and third dose of vaccines, respectively. CoronaVac was the most popular type, accounting for more than half of each dose. By contrast, mRNA vaccine (mRNA-1,273 (Moderna-NIAID)), viral vector-based vaccines (Ad26.COV2.S (Janssen), AD5-nCoV (CanSinoBio)), and protein subunit vaccine (ZF2001 (Anhui Zhifei Longcom)) were used on a relatively small scale (Figures 2D–F).

### Factors cross-sectionally associated with vaccination status

The survey participants were divided into two groups: the vaccinated group [1,459 cases (50.2%)] and the unvaccinated group [1,445 cases (49.8%)]. Table 1 shows the differences in the basic characteristics between the two groups. In the univariate model (Table 2), the vaccination status was significantly associated with monthly household income, work status, self-perceived health, recent breast cancer-related treatment, time after surgery, history of food or drug allergies, history of vaccine allergies, stage at diagnosis, former experience in consulting healthcare workers, and perceptions of vaccine protection or safety. However, age, educational attainment, administrative regions, rurality, having children under 18 years of age, histology, histological grade, molecular subtype of breast cancer, and history and worries about infection were not significantly associated with the vaccination status.

**Table 2:** Univariate and multivariate logistic regression of characteristics for association with vaccination status.

	Univariate logistic regression analysis	95% CI for OR			Multivariate logistic regression analysis^***^	95% CI for OR		
	OR	Lower	Upper	Value of *p*	OR	Lower	Upper	Value of *p*
**Sociodemographic variables**								
Age in years								
25–39	Ref.							
40–49	1.024	0.789	1.328	0.859				
50–59	0.926	0.715	1.198	0.558				
60–69	0.930	0.697	1.240	0.621				
70–79	0.713	0.472	1.074	0.107				
80+	0.523	0.249	1.056	0.076				
Missing^*^								
**Educational attainment**								
Undergraduate	Ref.							
Postgraduate	1.151	0.900	1.474	0.264				
High school and below	0.870	0.744	1.016	0.078				
**Monthly household income *per capita*, yuan**								
2,000–5,000	Ref.							
<2000	1.063	0.773	1.461	0.707				
5,000-10,000	1.173	0.981	1.403	0.081				
>10,000	1.282	1.062	1.548	0.010				
**Administrative regions**								
East	Ref.							
North	0.977	0.749	1.274	0.861				
Northeast	1.131	0.788	1.624	0.505				
Central	1.063	0.636	1.780	0.815				
South	2.090	0.894	5.291	0.100				
Southwest	0.605	0.232	1.489	0.283				
Northwest	0.894	0.512	1.558	0.693				
**Living area**								
Urban	Ref.							
Rural	1.023	0.765	1.369	0.879				
**Work status**								
Unemployed	Ref.				Ref.			
Employed	1.363	1.062	1.751	0.015	1.783	1.118	2.842	0.015
Retired	0.866	0.675	1.110	0.256	1.049	0.661	1.666	0.839
Student	2.121	0.201	45.916	0.541	390185542.547	0.000	.	1.000
**Have children under age 18**								
No	Ref.							
Yes	0.861	0.740	1.001	0.051				
**Health and disease status**								
**Self-perceived health**								
Bad	Ref.							
General	0.448	0.244	0.797	0.007				
Good	0.536	0.296	0.940	0.034				
**Recent breast cancer-related treatment**								
Cytotoxic therapy^**^	Ref.				Ref.			
Endocrine therapy	0.478	0.400	0.570	<0.001	0.531	0.376	0.749	<0.001
Traditional Chinese medicine	0.489	0.313	0.757	0.001	0.932	0.389	2.233	0.875
No treatment	0.858	0.697	1.055	0.147	1.124	0.745	1.693	0.578
Missing^*^								
**Time after surgery**								
<1 year	Ref.				Ref.			
1–3 years	0.263	0.210	0.329	<0.001	0.277	0.176	0.436	<0.001
3–5 years	0.292	0.228	0.373	<0.001	0.277	0.170	0.451	<0.001
> = 5 years	0.340	0.270	0.427	<0.001	0.282	0.179	0.443	<0.001
Missing^*^								
**History of food or drug allergies**								
No	Ref.				Ref.			
Yes	0.529	0.442	0.632	<0.001	0.579	0.417	0.804	0.001
**History of other vaccine allergies**								
No	Ref.							
Yes	0.317	0.211	0.467	<0.001				
**Stage at diagnosis**								
0	Ref.				Ref.			
I	3.214	2.255	4.598	<0.001	2.008	1.124	3.590	0.019
II	1.130	0.803	1.594	0.485	1.062	0.637	1.772	0.817
III	0.947	0.666	1.349	0.760	0.801	0.472	1.360	0.411
IV								
Missing^*^								
**Histology**								
Carcinoma *in situ*	Ref.							
Invasive ductal carcinoma	0.893	0.682	1.167	0.408				
Invasive lobular carcinoma	1.134	0.673	1.923	0.639				
Others	0.927	0.626	1.371	0.704				
Missing^*^								
**Histological grade**								
G1	Ref.							
G2	1.129	0.841	1.518	0.420				
G3	1.159	0.847	1.586	0.358				
Missing^*^								
**Molecular subtype**								
Luminal A	Ref.							
Luminal B	0.884	0.714	1.095	0.259				
HER2 over-expression subtype	0.945	0.657	1.360	0.762				
Basal-like	0.985	0.702	1.385	0.933				
Missing^*^								
**Variables related to COVID-19**								
**History of COVID-19 infection**								
No	Ref.							
Yes, no symptoms	0.991	0.039	25.078	0.995				
Yes, mild symptoms	0.991	0.183	5.363	0.991				
Yes, severe symptoms	1.321	0.291	6.719	0.716				
**Worried about infection**								
No	Ref.							
Yes	1.094	0.933	1.282	0.270				
**Have you consulted healthcare workers about COVID-19 vaccines?**								
No	Ref.							
Yes, my questions were answered.	1.035	0.881	1.215	0.676				
Yes, my questions were not answered.	0.803	0.651	0.989	0.039				
**Think vaccines can provide protection**								
No	Ref.				Ref.			
Yes	1.549	1.285	1.867	<0.001	1.774	1.170	2.690	0.007
**Perceptions on vaccine safety**								
General	Ref.				Ref.			
Safe	1.719	1.459	2.027	<0.001	2.074	1.513	2.843	<0.001
Very safe	2.963	2.229	3.967	<0.001	4.251	2.452	7.369	<0.001
Not safe	1.506	1.145	1.980	0.003	2.075	1.185	3.635	0.011
Very unsafe	5.024	3.038	8.699	<0.001	5.609	1.807	17.407	0.003

Values in red indicates these are statistically significant

OR, odds ratio; CI, confidence interval.

* Missing values were not included for statistical analysis.

** Chemotherapy/radiotherapy/targeted therapy, with/without endocrine therapy or traditional Chinese medicine.

*** Intercept = 0.15 (*p* = 0.722); Cox & Snell R Square = 0.192; Nagelkerke R Square = 0.256.

In the multivariable model (Table 2), the value of *p* for the Hosmer–Lemeshow test was 0.866, suggesting an acceptable fit. Self-perceived health, monthly household income, history of vaccine allergies, and former experience in consulting healthcare workers turned out to not significantly associate with the vaccination status. Employment was closely associated with vaccination status, compared with unemployment (OR = 1.783, 95% CI, 1.118–2.842, *p* = 0.015). The vaccination rate decreased for participants who had recently undergone endocrine therapy compared with those receiving cytotoxic therapy (OR = 0.531, 95% CI, 0.376–0.749, *p* < 0.001). Compared with less than 1 year after surgery, 1–3 years, 3–5 years, and more than 5 years significantly decreased the rate of vaccination (OR = 0.277, 95% CI, 0.176–0.436, *p* < 0.001; OR = 0.277, 95% CI, 0.170–0.451, *p* < 0.001, OR = 0.282, 95% CI, 0.179–0.443, *p* < 0.001). Participants with stage I disease at diagnosis were more likely to be vaccinated (OR = 2.008, 95% CI, 1.124–3.590, *p* = 0.019). Additionally, history of food or drug allergies significantly decreased the rate of vaccination (OR = 0.579, 95% CI, 0.417–0.804, *p* = 0.001).

As for perceptions, participants who thought vaccines could provide protection were more likely to be vaccinated than those who did not (OR = 1.774, 95% CI, 1.170–2.690, *p* = 0.007). Finally, participants who thought COVID-19 vaccines were safe (OR = 2.074, 95% CI, 1.513–2.843, *p* < 0.001), very safe (OR = 4.251, 95% CI, 2.452–7.369, *p* < 0.001), not safe (OR = 2.075, 95% CI, 1.185–3.635, *p* = 0.011), and very unsafe (OR = 5.609, 95% CI, 1.807–17.407, *p* = 0.003) showed higher vaccination rates than those who held general ideas (between safe and not safe).

### Side effects reported for different types of COVID-19 vaccines

**Figure 3:**
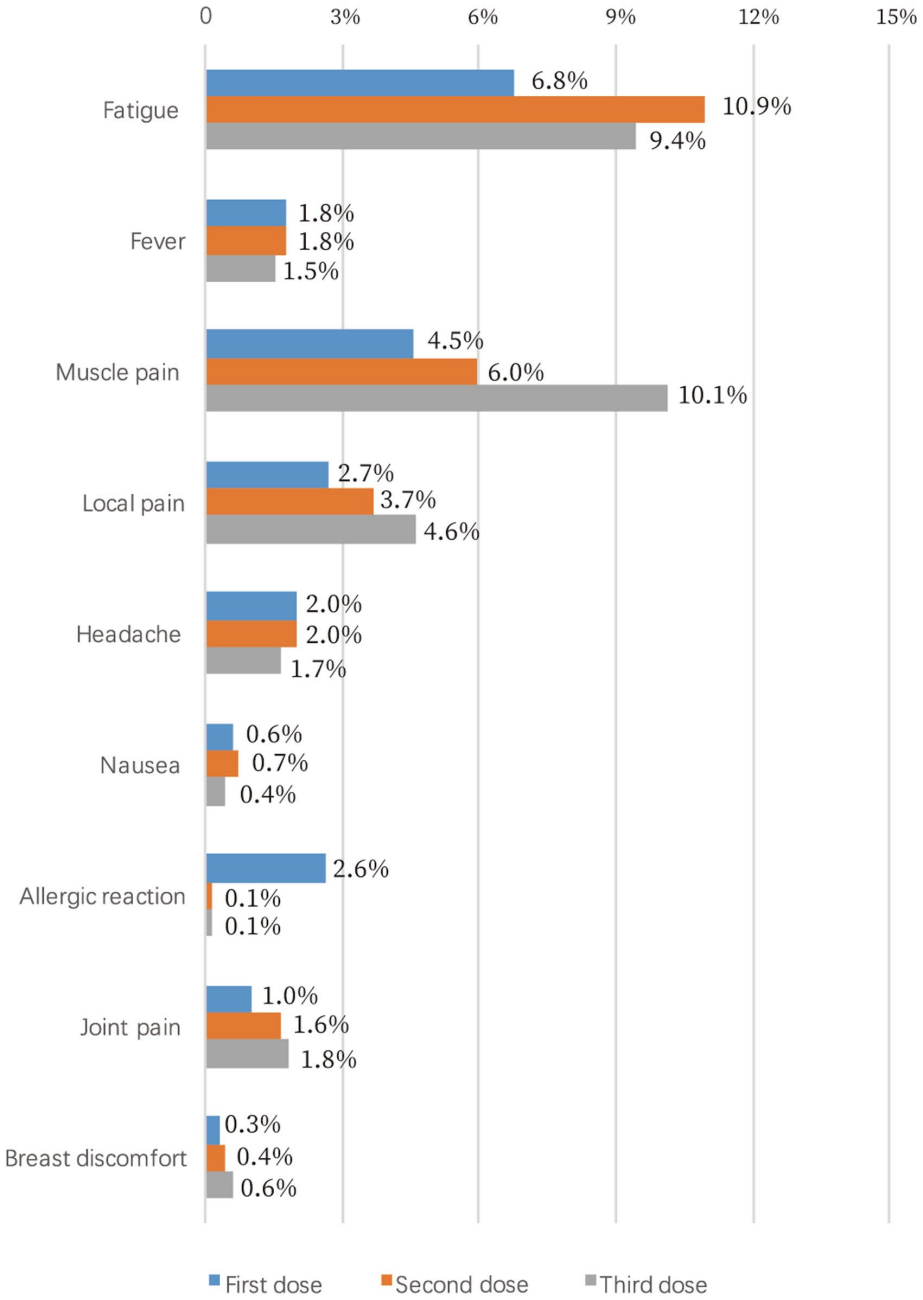
Side effects of COVID-19 vaccines.

The side effect rates for each vaccine dose are illustrated in Figure 3. Of the 1,459 vaccinated participants, 186 (12.7%) reported side effects after the first dose, including 99 cases (6.8%) of fatigue, 66 cases (4.5%) of muscle pain, and 38 (2.6%) cases of allergic reaction. The most common side effect for the second dose was fatigue, accounting for 10.9% of 1,407 participants, while muscle pain (73/720, 10.1%) was the most common side effect for the third dose. Notably, breast discomfort, described as breast itching, tenderness, swelling, or pain, was reported by 0.3–0.6% of the participants. The side effect rates among different types of COVID-19 vaccines were significantly different for first and the third dose (*p* = 0.007 and 0.019, respectively), whereas no difference was observed for the second dose (*p* = 0.169, Table 3). Pearson’s chi-squared test showed that the side effect rate was significantly increased if any previous COVID-19 vaccine dose led to side effects (*p* < 0.05).

**Table 3:** COVID-19 vaccine type and side effect rate.

	Total sample *N* (col%)	Sample with side effect *n* (col%)	*Value of p* ^**^
**Type of the first dose**	**1,459**	**186 (12.7)**	
BBIBP-CorV	561 (38.5)	78 (13.9)	0.007
CoronaVac	807 (55.3)	99 (12.3)	
WIBP-CorV	13 (0.9)	1 (7.7)	
AD5-nCoV	9 (0.6)	5 (55.6)	
ZF2001	50 (3.4)	1 (2.0)	
KCONVAC	2 (0.1)	0 (0.0)	
mRNA-1,273	5 (0.3)	0 (0.0)	
Ad26.COV2.S	1 (0.1)	0 (0.0)	
Sorry, I do not remember^*^	11 (0.8)	2 (18.2)	
**Type of the second dose**	**1,407**	**207 (14.7)**	
BBIBP-CorV	554 (39.4)	77 (13.9)	0.169
CoronaVac	770 (54.7)	121 (15.7)	
WIBP-CorV	14 (1.0)	4 (28.6)	
AD5-nCoV	4 (0.3)	1 (25.0)	
ZF2001	48 (3.4)	3 (6.3)	
KCONVAC	3 (0.2)	0 (0.0)	
mRNA-1,273	3 (0.2)	1 (33.3)	
Sorry, I do not remember^*^	11 (0.8)	0 (0.0)	
**Type of the third dise**	**720**	**101 (14.0)**	
BBIBP-CorV	270 (37.5)	38 (14.1)	0.019
CoronaVac	378 (52.5)	50 (13.2)	
WIBP-CorV	2 (0.3)	1 (50.0)	
AD5-nCoV	4 (0.6)	3 (75.0)	
ZF2001	57 (7.9)	8 (14.0)	
KCONVAC	1 (0.1)	0 (0.0)	
mRNA-1,273	2 (0.3)	0 (0.0)	
IMBCAMS	1 (0.1)	1 (100.0)	
Sorry, I do not remember^*^	6 (0.8)	0 (0.0)	

* Not included for statistical analysis.

** Results from Fisher’s exact test.Bold values are the sums for each dose

### Willingness to receive another dose of COVID-19 vaccine

**Figure 4:**
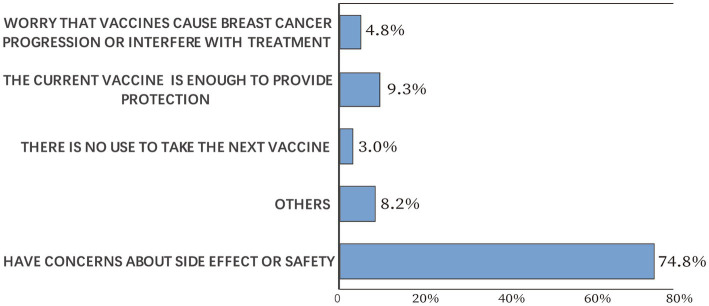
Participants’ reasons for not taking another COVID-19 vaccine dose.

Participants’ willingness to receive another dose of COVID-19 vaccine was explored among the vaccinated cases. Of the 1,459 participants, 639 (43.8%) would accept another vaccine dose. Participants’ reasons for not taking another COVID-19 vaccine dose are illustrated in Figure 4. The most common reason was “have concerns about side effects or safety” (74.8%), followed by “the current vaccine is enough to provide protection” (9.3%). Only 4.8% of the vaccinated participants worried that vaccines would cause breast cancer progression or interfere with treatment, and 3.0% of the vaccinated participants thought there was no use to take the next dose. According to Pearson’s chi-squared test, participants’ willingness to receive another vaccine dose was significantly decreased if they experienced COVID-19 vaccine side effects (*p* < 0.05).

## Discussion

In the Chinese population-based survey study, we used a quota-sampled method to recruit a total of 2,904 patients with breast cancer who had undergone breast surgery at PUMCH. COVID-19 vaccination coverage rates, side effects, concerns and perceptions were assessed, along with other relevant variables. People who were administered with the complete protocol, first dose, and booster dose in the Chinese mainland accounted for 89.7, 92.1, and 71.7% of the total population, respectively (23). By contrast, our results revealed relatively lower rates of complete-protocol administration (24.8%) and first-dose administration (50.2%) among breast cancer survivors in the Chinese mainland. The finding underscores the importance of promoting COVID-19 vaccination among patients with breast cancer. More importantly, we sought to find reasons underlying the vaccination rate gap between breast cancer survivors and the general population, and customize strategies to improve the vaccination rate in cancer population.

A major concern for COVID-19 vaccination is safety. Our results indicated that more than half of the vaccinated cases had concerns about side effects, which accounted for nonvaccination in 39.6% of the unvaccinated cases. What’s more, 74.8% of the vaccinated patients did not want to receive another dose of COVID-19 vaccine mainly because of safety concerns. This is consistent with results from the general population and other population groups (24–27). According to a survey study in Poland, 49.2% of the participants refused to receive a booster dose because of side effects experienced after previous doses, and 22.4% because of safety uncertainties (28). In addition, a recent study among university students in Egypt revealed that the main reason for vaccine hesitation was being afraid of serious side effects (29).

In reality, the safety profiles of COVID-19 vaccines reported by our study are largely acceptable. The side effect rates are comparable to those of inactivated virus vaccines in the general population (30, 31), and noticeably better than those of mRNA vaccines in cancer patients (9, 32, 33). A cohort study of 160 breast cancer patients in Iran who received BBIBP-CorV showed that the most common local and systemic side-effects were injection site pain and fever, accounting for 22.3 and 24.3% of the patients, respectively (34). While our results showed that the most common local and systemic side-effects were local pain and fatigue, accounting for 2.7–4.6% and 6.8–10.9% of the patients, respectively. Because many clinical trials on COVID-19 vaccines excluded patients with malignancies, the report of our findings would help reduce vaccine hesitation.

Meanwhile, disease-related concerns cannot be overestimated in vaccination behaviors. 72.9% of the participants did not receive COVID-19 vaccines because they worried that vaccines would cause breast cancer progression or interfere with treatment, and 35.3% of the vaccinated cases were primarily concerned that vaccines would cause breast cancer progression. Although long-term follow-ups remain unavailable, results from our study indicate low rates (0.3–0.6%) of breast discomfort following vaccination. Besides, axillary lymphadenopathy, which could be a clinical manifestation of ipsilateral breast cancer progression, was more commonly reported in cases who received mRNA vaccines (0.1–16%) (35), and most inactivated virus vaccines did not document axillary lymphadenopathy as a solicited adverse event (36–38).

In our study, recent breast cancer-related treatment, time after surgery, and stage at diagnosis were found to be independently related to vaccination status. We found that patients who recently underwent endocrine therapy were less likely to take COVID-19 vaccines. And patients who were less than 1 year after surgery or at stage I were more likely to receive vaccination, probably because there was no ongoing adjuvant treatment. Some participants reported that doctors asked them to wait for 6 months to 3 years after systematic therapies before vaccines. As far as we know, this criterion was extensively used in China in 2020 and early 2021, when COVID-19 vaccines initially came to market with limited safety results in cancer population (30, 36). In late 2021, the vaccination criterion became obscure following more experience gained in breast cancer patients (39). However, it is of note that the inconsistency of contraindications would cause confusion and vaccine hesitancy, and 8.8% of the participants were not vaccinated because they would like to wait for further results. Because fragmented reports and biased information could foster vaccine hesitancy (40), it is imperative for the government and health institutions to launch educational campaigns to provide breast cancer survivors with adequate information on the precautions, indications, contraindications, and potential side effects of COVID-19 vaccines.

Efficacy (protection) is a driving force for vaccination. Compared with the unvaccinated group, a significantly larger proportion of the vaccinated group thought vaccines could provide protection (77.4 vs. 84.2%). The rates are in parallel with those of the general population (29, 41). Over half of the participants got vaccinated because of “fear of infection,” and nearly 10% of the vaccinated participants did not want to receive the next dose because they believed the current vaccine was enough to provide protection. However, unlike healthy individuals, the low seropositive rate of vaccine-induced antibodies in patients with malignancies indicates a lack of virus-neutralizing activity and justifies the use of booster doses (10, 42, 43). A better understanding of their vulnerability to COVID-19 and potential immunosenescence to vaccination would help facilitate periodic vaccination in patients with breast cancer. To evaluate the efficacy of COVID-19 boosters in patients with breast cancer, our research team is currently investigating the immunogenicity and immune response following COVID-19 vaccines in breast cancer cohorts.

To accelerate COVID-19 vaccination and tackle healthcare inequities, the Chinese government has implemented a series of robust measures. Resources from around the nation were galvanized for vaccine development and adequate domestic production capacity (44). As of July 30, 2022, more than 3.4 billion doses of COVID-19 vaccines had been administered in China (45). Till now, seven types of domestically developed vaccines have been offered free of charge to the public, including five inactivated virus vaccines (IMBCAMS, KCONVAC, BBIBP-CorV, CoronaVac, WIBP-CorV), one protein subunit vaccine (ZF2001), and one adenovirus vaccine (AD5-nCoV) (46). Results from our study show that inactivated virus vaccines led the Chinese COVID-19 vaccine market in patients with breast cancer. Additionally, the local governments have undertaken plenty of measures to stimulate vaccination, including setting up temporary inoculation points and extending the service hours of inoculation sites. Vaccines were offered door-to-door for certain works and for those with poor spatial accessibility or mobility. The study shows that administrative regions, household income, and having children under 18 years of age were comparable between the vaccinated and unvaccinated groups. Only 1.0% of the participants did not receive vaccination because of difficulties in reaching vaccination institutions. Of note, work status was significantly associated with vaccination status in the univariate and multivariate analyses. In fact, approximately one-third of the participants reported receiving vaccination mainly because of workplace or government requirement. In this context, future vaccination promotion should particularly target at the unemployed.

This study has strengths and limitations. The cross-sectional survey design enabled a swift collection of valuable, real-world data on the ever-evolving COVID-19 pandemic. Our strengths are the large sample size and representativeness of the sample. Importantly, the quota-sampled approach achieved expected distributions with respect to age and years after surgery. However, because of the single-center design, the study failed to achieve equalized distributions of certain sociodemographic variables, such as educational attainment, administrative regions, and living area, even though these variables were not associated with vaccination status according to the results from univariate and multivariate analyses. Moreover, this study managed to assess valuable pathological records and clinical stage in around 60–80% of the participants. Also, the questionnaire was piloted, enabling its capacity to cover appropriate questions. For example, breast discomfort was not *a priori* defined as one of the multiple choices of side effects, but it was decided to be an independent choice after discussion by specialists accessing the pilot results. Consequently, the survey could, to a large extent, avoid misleading and underreporting. We provided valuable records of the side effects of COVID-19 vaccines. However, we did not collect data on the time and severity of side effects. These and other unmeasured variables (e.g., chronic disease history) could cause residual confounding or bias, which might have skewed our results. Finally, though we applied multiple methods to avoid inaccuracy of self-reported information (e.g., information attainment and validation with HIS, asking participants to check their vaccine records), the use of an online questionnaire might have an influence on information validity.

In conclusion, this study suggests an overall need for vaccination promotion among Chinese breast cancer patients. Vaccination could be promoted by stressing the importance of periodic vaccination in cancer patients, and increasing confidence in vaccine safety during breast cancer treatment. Efforts should be particularly focused on the unemployed individuals.

## Data availability statement

The raw data supporting the conclusions of this article will be made available by the authors, without undue reservation.

## Ethics statement

The studies involving human participants were reviewed and approved by Peking Union Medical College Hospital. The informed consent of the survey was implied by completing the online survey.

## Author contributions

QS and YoL: conceptualization and supervision. LL and YX: investigation and data cleaning. LL: methodology, visualization, and writing. LL, YX, XL, HL, YS, YLiu, CC, HZ, ZW, XF, ML, YW, GL, YuL, and YQ: data collection. QS and YoL: funding acquisition. All authors contributed to the article and approved the submitted version.

## Funding

This work was supported by the Chinese Academy of Medical Sciences Innovation Fund for Medical Sciences (CIFMS 2021-I2M-1-014), National Key Research and Development Program of China (2018YFE0207300), Beijing Natural Science Foundation (M23008), and Beijing Municipal Science & Technology Commission (Z211100002521021).

## Conflict of interest

The authors declare that the research was conducted in the absence of any commercial or financial relationships that could be construed as a potential conflict of interest.

## Publisher’s note

All claims expressed in this article are solely those of the authors and do not necessarily represent those of their affiliated organizations, or those of the publisher, the editors and the reviewers. Any product that may be evaluated in this article, or claim that may be made by its manufacturer, is not guaranteed or endorsed by the publisher.

## Footnotes

^1^ https://www.wjx.cn
