# Supplementary material for: COVID-19 vaccination status, side effects, and perceptions among breast cancer survivors: a cross-sectional study in China
Source: Front Public Health. 2023 Apr 17;11:1119163. doi: 10.3389/fpubh.2023.1119163 (PMC10150050; doi:10.3389/fpubh.2023.1119163)
Supplement: Supplementary file 1 [file Data_Sheet_1.PDF]

Survey Questionnaire: Breast Cancer Patients' COVID-19 Vaccination Status and Perceptions (English version)

1. Your registration ID at Peking Union Medical College Hospital: [Fill in the blank]

2. Have you ever been infected with COVID-19? [Single choice]
○ No
○ Yes, I had no symptoms
○ Yes, I had mild symptoms
○ Yes, I had severe symptoms

3. How would you describe your current health condition? [Single choice]
○ Good
○ General
○ Bad

4. What is your recent breast cancer-related treatment? [Multiple choice]
☐ Chemotherapy
☐ Endocrine therapy
☐ Radiotherapy
☐ Targeted therapy
☐ Traditional Chinese medicine
☐ None
☐ Other: ________________

5. Have you received a COVID-19 vaccine? [Single choice]
○ Not vaccinated
○ Received one dose
○ Received two doses
○ Received three doses

6. What was the main reason for you to receive the COVID-19 vaccine? [Single choice]
○ Workplace/government requirement
○ Fear of infection
○ Doctor's advice
○ Family/friend's recommendation
○ Other: ________________
Depends on the second, third, and fourth option of question 5.

7. What are the reasons that you have not received a COVID-19 vaccine? [Multiple choice]
○ Have concerns about side effects or safety
○ Cannot reach vaccination institutions
○ Don't think I will be infected
○ Don't think it would be serious even if infected

○ Would like to wait for further results
○ Have concerns about vaccine effectiveness
○ Fear of needles
○ Worry that vaccines cause breast cancer progression or interfere with treatment
○ Other:_________________
Depends on the first option of question 5.

8. Do you have a history of food or drug allergies? [Single choice]
○ Yes
○ No

9. Have you experienced allergic reactions when receiving other vaccines? [Single choice]
○ Yes
○ No

10. What was the main concern for you before receiving the COVID-19 vaccine? [Single choice]
○ Vaccine effectiveness
○ The vaccine could cause breast cancer progression
○ Other side effects
○ Nothing special
Depends on the second, third, and fourth option of question 5.

11. Type of the first dose [Single choice]
○ Sinopharm (Beijing)
○ CoronaVac/SinoVac
○ Sinopharm/WIBP
○ Convidecia/CanSinoBio
○ Zhifei Longcom, China
○ KCONVAC
○ BioNTech-Pfizer/BNT162b2
○ Sorry, I don't remember
○ Other: _________________
Depends on the second, third, and fourth option of question 5.

12. Date of the first dose of vaccination [Fill in the blank]
Depends on the second, third, and fourth option of question 5.

13. Did you experience adverse reactions after receiving the first dose? [Single choice]
○ Yes
○ No (Please skip to question 15)
Depends on the second, third, and fourth option of question 5.

14. Adverse reactions experienced after receiving the first dose [Multiple choice]
☐ Fatigue

☐ Fever
☐ Muscle pain
☐ Local pain
☐ Headache
☐ Nausea
☐ Allergic reaction
☐ Joint pain
☐ Breast discomfort
☐ Other: ________________
Depends on the second, third, and fourth option of question 5.

15. Type of the second dose [Single choice]
○ Sinopharm (Beijing)
○ CoronaVac/SinoVac
○ Sinopharm/WIBP
○ Convidecia/CanSinoBio
○ Zhifei Longcom, China
○ KCONVAC
○ BioNTech-Pfizer/BNT162b2
○ Sorry, I don't remember
○ Other: ________________
Depends on the third and fourth option of question 5.

16. Date of the second dose of vaccination [Fill in the blank]
Depends on the third and fourth option of question 5.

17. Did you experience adverse reactions after receiving the second dose? [Single choice]
○ Yes
○ No (Please skip to question 19)
Depends on the third and fourth option of question 5.

18. Adverse reactions experienced after receiving the second dose [Multiple choice]
☐ Fatigue
☐ Fever
☐ Muscle pain
☐ Local pain
☐ Headache
☐ Nausea
☐ Allergic reaction
☐ Joint pain
☐ Breast discomfort
☐ Other: ________________
Depends on the third and fourth option of question 5.

19. Type of the third dose [Single choice]
○ Sinopharm (Beijing)
○ CoronaVac/SinoVac
○ Sinopharm/WIBP
○ Convidecia/CanSinoBio
○ Zhifei Longcom, China
○ KCONVAC
○ BioNTech-Pfizer/BNT162b2
○ Sorry, I don't remember
○ Other: _________________
Depends on the fourth option of question 5.

20. Date of the third dose of vaccination [Fill in the blank]
Depends on the fourth option of question 5.

21. Did you experience any adverse reactions after receiving the third dose? [Single choice]
○ Yes
○ No (Please skip to question 23)
Depends on the fourth option of question 5.

22. Adverse reactions experienced after receiving the third dose [Multiple choice]
☐ Fatigue
☐ Fever
☐ Muscle pain
☐ Local pain
☐ Headache
☐ Nausea
☐ Allergic reaction
☐ Joint pain
☐ Breast discomfort
☐ Other: _________________
Depends on the fourth option of question 5.

23. Are you willing to receive another dose of COVID-19 vaccine? [Single choice]
○ Yes
○ No
Depends on the second, third, and fourth option of question 5.

24. Why are you unwilling to receive another dose of COVID-19 vaccine? [Single choice]
○ There is no use to take the next vaccine
○ Have concerns about side effect or safety
○ The current vaccine is enough to provide protection
○ Other: _________________
Depends on the second option of question 23.

25. Your current place of residence [Single choice]
○ East China region (Shandong, Jiangsu, Anhui, Zhejiang, Fujian, Shanghai)
○ South China region (Guangdong, Guangxi, Hainan)
○ Central China region (Hubei, Hunan, Henan, Jiangxi)
○ North China region (Beijing, Tianjin, Hebei, Shanxi, Inner Mongolia)
○ Northwest China region (Ningxia, Xinjiang, Qinghai, Shaanxi, Gansu)
○ Southwest China region (Sichuan, Yunnan, Guizhou, Tibet, Chongqing)
○ Northeast China region (Liaoning, Jilin, Heilongjiang)
○ Other (overseas): _________________

26. Your current living area [Single choice]
○ Rural
○ Urban

27. Your current work status [Single choice]
○ Unemployed
○ Employed
○ Retired
○ Student

28. Are you worried about COVID-19 infection? [Single choice]
○ Yes
○ No

29. Do you believe that vaccines can prevent COVID-19? [Single choice]
○ Yes
○ No

30. What do you think about the safety of current COVID-19 vaccines? [Single choice]
○ Very safe
○ Safe
○ General
○ Not safe
○ Very unsafe

31. Have you consulted healthcare workers about COVID-19 vaccines? [Single choice]
○ Yes
○ No

33. Do you think your questions were answered? [Single choice]
○ Yes
○ No
Depends on the first option in question 31.

34. What is your education level? [Single choice]

○ High school and below

○ Undergraduate

○ Postgraduate

35. What is your family's average monthly income per capita (yuan)? [Single choice]

○ 2,000-5,000

○ <2000

○ 5,000-10,000

○ >10,000

36. Do you have any children under 18 years old? [Single choice]

○ Yes

○ No

37. What was the date of your surgery? [Fill in the blank]
