# Supplementary material for: COVID-19 vaccination status, side effects, and perceptions among breast cancer survivors: a cross-sectional study in China
Source: Front Public Health. 2023 Apr 17;11:1119163. doi: 10.3389/fpubh.2023.1119163 (PMC10150050; doi:10.3389/fpubh.2023.1119163)
Supplement: Supplementary file 2 [file Data_Sheet_2.PDF]

Supplementary figures: Comparisons of quota-sampled participants and national population with breast cancer

Supplementary figure 1. Comparisons of the age distributions of quota-sampled participants vs national breast cancer population by prevalence

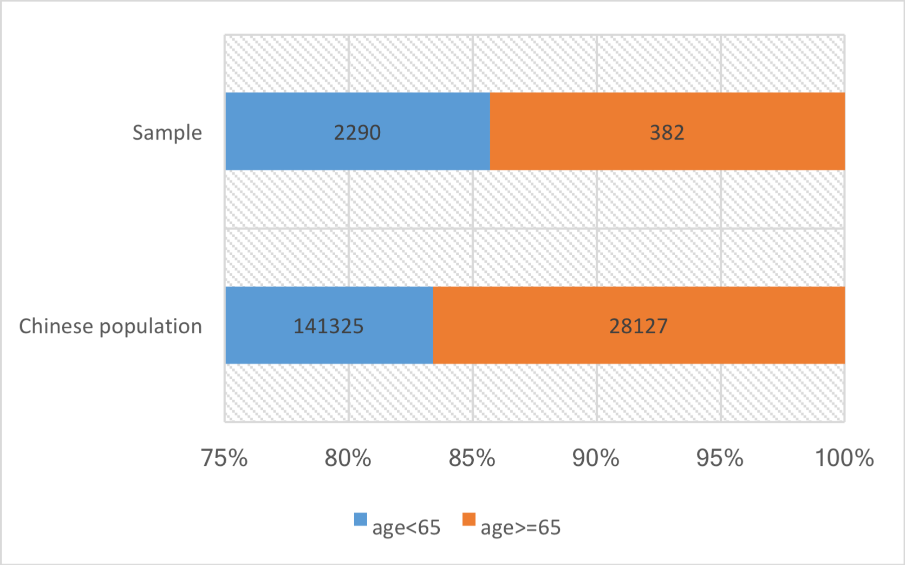

Source for the Chinese breast cancer age distribution by prevalence: age distributions in China in 2008 based on data from the WHO China country profile (WHO. China country profile 2011, http://www.wpro.who.int/countries/chn/5CHNpro2011_finaldraft.pdf (accessed June 21, 2013)[1].

The prevalence of breast cancer is rarely recorded or reported in China, resulting in extremely limited statistics[2]. The prevalence of national breast cancer population age distributions was only available and demonstrated by two age groups in Supplementary figure 1.

Supplementary figure 2. Comparisons of the age distributions of quota-sampled participants vs national breast cancer population by incidence

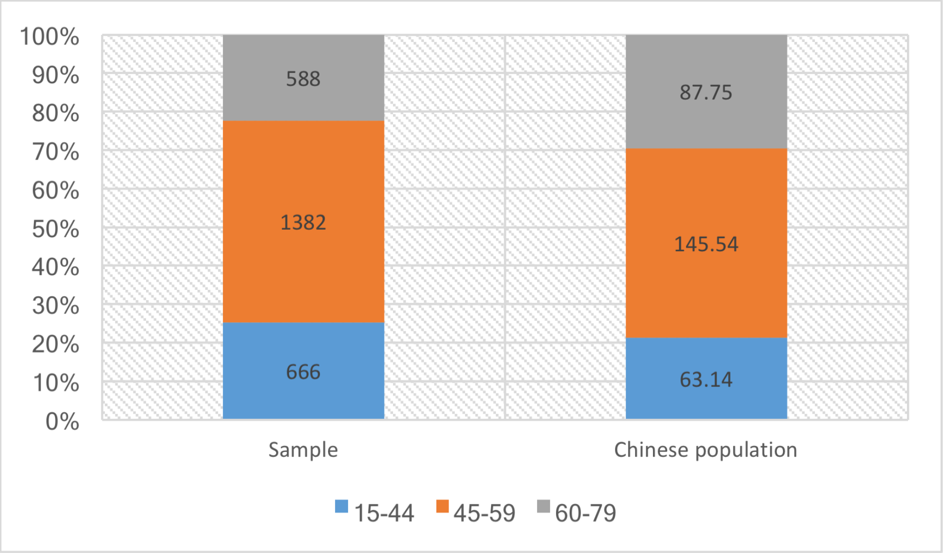

Source for the Chinese breast cancer age distribution by incidence: estimated numbers of new cancer cases of all cancers and five leading cancer types by age in China, 2016 (registration data of 2016 submitted by a total of 682 cancer registries from 31 provinces, autonomous regions and municipalities and Xinjiang Production and Construction Corps to Chinese National Cancer Center)[3]. The shifting of incidence peaked to older age groups during the last three decades[2].

Supplementary figure 3. Distributions of the time after surgery

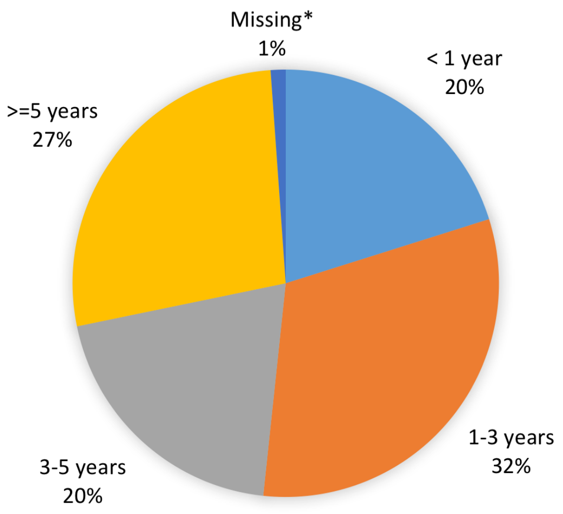

The numbers of participants who were >=5 years, 3-5 years, 1-3 years, and < 1 years after surgery were balanced.

Supplementary figure 4. Comparisons of the regional distributions of quota-sampled participants vs national breast cancer population

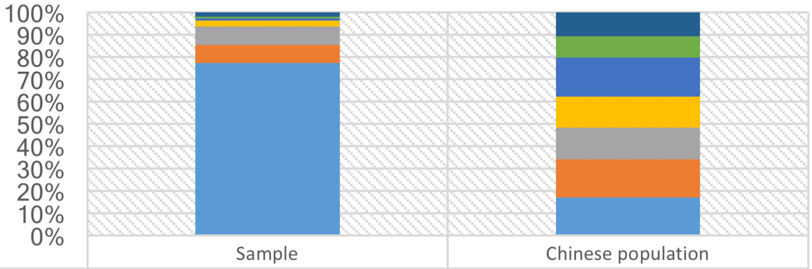

	Sample	Chinese population
Northwest	63	21.6
Southwest	21	18.6
South	25	34.8
Central	77	28
East	242	27.7
Northeast	230	34.1
North	2246	34

Source for the Chinese breast cancer regional distributions: age-standardized incidence rates overall, by area and cancer type by geographic areas covered by 487 cancer registries, 2016. (registration data of 2016 submitted by a total of 682 cancer registries from 31 provinces, autonomous regions and municipalities and Xinjiang Production and Construction Corps to Chinese National Cancer Center)[3].

Supplementary figure 5. Comparisons of the type by geographic areas of quota-sampled participants vs national breast cancer population

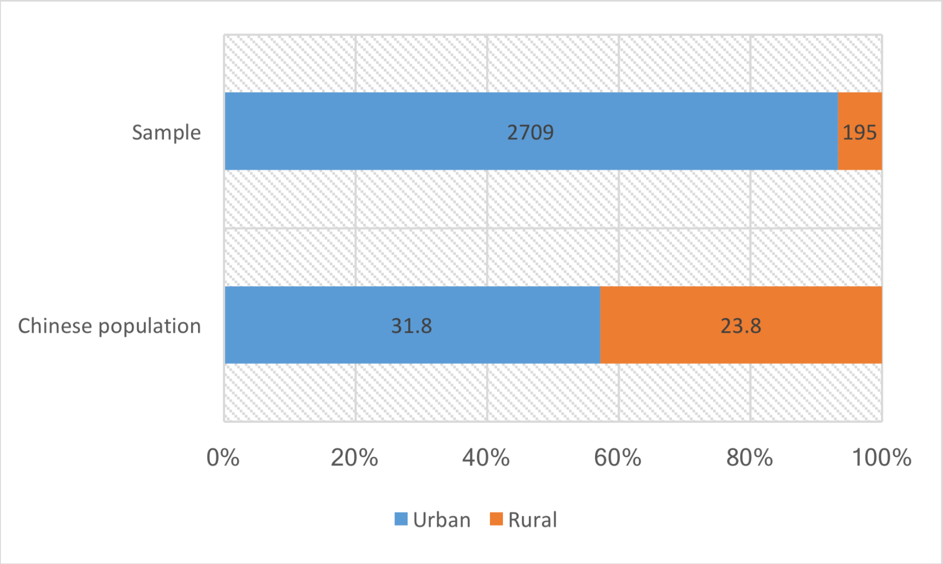

Source for the Chinese breast cancer regional distributions: age-standardized incidence rates overall, by area and cancer type by geographic areas covered by 487 cancer registries, 2016. (registration data of 2016 submitted by a total of 682 cancer registries from 31 provinces, autonomous regions and municipalities and Xinjiang Production and Construction Corps to Chinese National Cancer Center)[3].

References:

1 Fan L, Strasser-Weippl K, Li J-J, *et al.* Breast cancer in China. *The Lancet Oncology* 2014; 15: e279–89.

2 Li T, Mello-Thoms C, Brennan PC. Descriptive epidemiology of breast cancer in China: incidence, mortality, survival and prevalence. *Breast Cancer Res Treat* 2016; 159: 395–406.

3 Zheng R, Zhang S, Zeng H, *et al.* Cancer incidence and mortality in China, 2016. *Journal of the National Cancer Center* 2022; 2: 1–9.
